# Beyond trial-and-error: Individualizing therapeutic transcranial neuromodulation for chronic pain

**DOI:** 10.1002/ejp.2164

**Published:** 2023-08-19

**Authors:** Daniel Ciampi de Andrade, Luís García-Larrea

**Affiliations:** 1 Department of Health Science and Technology, Faculty of Medicine, Center for Neuroplasticity and Pain (CNAP), Aalborg University, Aalborg, Denmark; 2 University Hospital Pain Center (CETD), Neurological Hospital P. Wertheimer, Hospices Civils de Lyon, Lyon, France; 3 NeuroPain Lab, INSERM U1028, UMR5292, Lyon Neuroscience Research Center, CNRS, University Claude Bernard Lyon 1, Lyon, France

## Abstract

**Background and Objective:**

Repetitive transcranial magnetic stimulation (rTMS) applied to the motor cortex provides supplementary relief for some individuals with chronic pain who are refractory to pharmacological treatment. As rTMS slowly enters treatment guidelines for pain relief, its starts to be confronted with challenges long known to pharmacological approaches: efficacy at the group-level does not grant pain relief for a particular patient. In this review, we present and discuss a series of ongoing attempts to overcome this therapeutic challenge in a personalized medicine framework.

**Databases and Data Treatment:**

Relevant scientific publications published in main databases such as PubMed and EMBASE from inception until March 2023 were systematically assessed, as well as a wide number of studies dedicated to the exploration of the mechanistic grounds of rTMS analgesic effects in humans, primates and rodents.

**Results:**

The main strategies reported to personalize cortical neuromodulation are: (i) the use of rTMS to predict individual response to implanted motor cortex stimulation; (ii) modifications of motor cortex stimulation patterns; (iii) stimulation of extra-motor targets; (iv) assessment of individual cortical networks and rhythms to personalize treatment; (v) deep sensory phenotyping; (vi) personalization of location, precision and intensity of motor rTMS. All approaches except (i) have so far low or moderate levels of evidence.

**Conclusions:**

Although current evidence for most strategies under study remains at best moderate, the multiple mechanisms set up by cortical stimulation are an advantage over single-target ‘clean’ drugs, as they can influence multiple pathophysiologic paths and offer multiple possibilities of individualization.

**Significance:**

Non-invasive neuromodulation is on the verge of personalised medicine. Strategies ranging from integration of detailed clinical phenotyping into treatment design to advanced patient neurophysiological characterisation are being actively explored and creating a framework for actual individualisation of care.

## 1 Repetitive Transcranial Magnetic Stimulation for Pain: Why and How

Neuromodulation refers to a long list of interventions leading to modifications in spontaneous neural activity, plasticity or information processing in the central or peripheral nervous system (Moisset et al., 2016). Defined in such broad terms, countless interventions may be called ‘neuromodulatory’, from taking a bath or listening to music, to attending a psychotherapy session or using psychoactive medication. Despite this wide definition, neuromodulation is a term most often used to refer to interventions where external physical agents (e.g. images, sounds or ultrasounds, electric currents) are delivered to the nervous system for therapeutic purposes (Moisset et al., 2020). The most used neuromodulatory agents are electric currents, which can be applied either directly upon nervous structures in the spinal cord, dorsal ganglion or the brain or externally, over the skin or scalp, as is the case of transcutaneous electric nervous stimulation (TENS) or transcranial direct current stimulation (tDCS) (Fernandes et al., 2022; Garcia-Larrea & Quesada, 2022). Such electric currents may also be induced indirectly, and painlessly, by a rapidly oscillating magnetic pulse, such as in transcranial magnetic stimulation (TMS). TMS is based on Faraday’s principle of electromagnetic induction: a very fast electric current flowing for 1 ms through a wiring system in a coil creates a powerful electromagnetic field (approximately 1 Tesla, Neumann, 1846; Barker et al., 1985; Faraday & Day, 1999), which in turn can induce a secondary electric current in a conductor a few centimetres away from the coil surface. In the case of TMS, the magnetic field is applied painlessly to the scalp and cortical axons are the electrical conductors receiving the induced electric current. TMS was originally designed as a neurophysiological tool to explore the corticomotor pathways and for performing mapping of selective cortical functions non-invasively (Barker et al., 1985; Bickford et al., 1987). It was soon realized that when TMS was delivered in repetitive pulses to the motor cortex, it could induce changes in corticospinal excitability which lasted beyond the stimulation period (Pascual-Leone et al., 1994). The discovery that the direction of these modifications towards increases or decreases of corticospinal excitability depended on the frequency of stimulation (Pascual-Leone et al., 1994), led to the development of repetitive TMS (rTMS) as a non-invasive modality of therapeutic neuromodulation, which was granted FDA approval for clinical use in 2008 in the USA (Foy, 2011, CFM. Resolution, 2012).Therapeutic rTMS has been applied since then with varying results, essentially for the treatment of major depression (focused on the dorsolateral prefrontal cortex) (Kan et al., 2023) auditory hallucinations (applied to the temporal lobes) (Kennedy et al., 2018; Marzouk et al., 2020) and chronic neuropathic pain (applied to the motor cortex; Garcia-Larrea & Quesada, 2022). In this review, we concentrated exclusively on rTMS use in the chronic pain domain, in particular in neuropathic pain. Our main aim is to discuss the rationale and grounds for a personalized tailoring of stimulation parameters to better meet the needs of people with chronic pain, and neuropathic pain was chosen as a model because a larger number of studies exist for this condition. Other pain types are mentioned when relevant to the discussion of methodological and technical aspects of rTMS for chronic pain in general.

## 2 Historical Perspective and Mechanisms of Rtms

In the pain field, rTMS was initially used to predict the effects of surgically implanted epidural motor cortex stimulation (iMCS), which was described as an analgesic procedure for neuropathic pain relief by Tsubokawa et al. (1991, 1993). Pioneers in rTMS use soon realized that beyond its effects as a predictor of iMCS efficacy, rTMS to the motor cortex had analgesic effects that went beyond the stimulation session and could be maintained by daily or even monthly iteration of rTMS sessions (Lefaucheur et al., 2001; Khedr et al. 2005). This opened the path to explore rTMS as a therapeutic modality of its own in the chronic pain field. In parallel, an intense ‘back–translational’ movement commenced early in this century, which explored the potential mechanistic effects (metabolic, hemodynamic and electrophysiological) of repetitive motor cortex stimulation delivered either epidurally or transcranially in both humans and experimental animal models.

Similar to epidural stimulation, local metabolic changes intrinsic to motor networks are scarce or absent during subthreshold rTMS stimulation (Bestmann et al., 2004; Siebner et al., 2000; Speer et al., 2003), whereas the notion that M1 rTMS preferentially influences areas far from the primary motor cortex has received consistent support. Abundant human data suggest that M1 rTMS influences a brain network that includes the anterior cingulate, insular and dorsolateral prefrontal cortices (DLPFC), striatum and brainstem (Bestmann et al., 2004; Hodkinson et al., 2021; Siebner et al., 2000). In rats and mice rTMS induced c-fos neural activation in regions distant from the stimulation site including thalamus, ACC, striatum and hippocampus (Doi et al., 2001; Hausmann et al., 2000; Ji et al., 1998) and rTMS-related functional connectivity changes between these areas has been described in both human patients and a nonhuman primate model of central pain (Kadono et al., 2021; Pei et al., 2019). It was recently shown that the primary motor cortex in macaques and humans is not a monotonous strip of motor effector areas, but contains instead non-motor islands with markedly distinct connectivity and structure, intermingled between the traditional foot, hand and mouth motor output areas. These non-motor areas are hubs connecting M1 to the cingular and operculo-insular cortices and are believed to play major roles in the bodily preparation for motor response by coupling it to ongoing interoceptive processing and visceromotor programs, arousal tonus, prediction error and pain. Additionally, these interpolated non-motor effector areas are highly interconnected between them, providing the basis for whole-body movement control (Gordon et al., 2023). Taken together these results suggest the view that M1 is a privileged ‘entry gate’ into the brain, allowing modulation of neuronal activity taking place in areas distant from, but functionally connected to M1 (Gan et al., 2022; Lefaucheur et al., 2014).

RTMS additionally engages changes in neurotransmitter endogenous opioid function. The contribution of mu-opioid receptors to the analgesic rTMS effects is supported by (i) enhancement of serum beta-endorphin after successful stimulation (Ahmed et al., 2011), (ii) naloxone blockade of rTMS analgesia (de Andrade et al., 2011) and (iii) rTMS-induced increase in brain opioid receptor occupancy (Lamusuo et al., 2017). Contribution of glutamate n-methyl-d-aspartate receptors received support from two studies in humans (Fregni et al., 2011; Ciampi de Andrade et al., 2014), while in rats rTMS effects were not blocked by NMDA antagonists (Hausmann et al., 2000).

The profile of cortical and subcortical structures influenced by rTMS to M1 may be dependent on the fibres preferentially stimulated within the precentral gyrus. A latero-medial orientation of the TMS coil, which optimizes the activation of the descending corticospinal tract, did not lead to significant analgesic effects, which were, on the contrary, attained when the coil was placed in the postero-anterior direction (André-Obadia et al., 2008). This is believed to depend on the preferential depolarization of cortico-cortical fibres in the postero-anterior coil orientation, thus influencing M1 outputs to other brain areas rather than to the descending pyramidal tract (Hodkinson et al., 2021). A recent experimental study using chemo-and optogenetics supports this view and suggests that M1 stimulation reaching deeper cortical column layers (VI) would influence emotional and coping behaviours in neuropathic pain models via a connection to the nucleus accumbens reward circuitry passing through the thalamus, which would be qualitatively different from the effects restricted to somatosensory pain components when layer V is preferentially stimulated (Gan et al., 2022).

## 3 Current Evidence and Current Limitations of Rtms Use for Chronic Pain

The assessment of rTMS efficacy for chronic pain has been traditionally hampered by the low quality of evidence due to small patient samples, absent blinding, lack of follow-up, no report on withdrawals and so forth. In 2018, a report from the Cochrane institution concluded that there was ‘low-quality evidence’ of positive rTMS effects on chronic pain, up to 6 weeks post-intervention (O’Connell et al., 2018). Since then, several well-conducted studies have been added to the literature, which now counts at least 14 randomized, placebo-controlled trials (RCTs) on rTMS in neuropathic pain, each reporting data from at least 20 patients in the active group and totaling ~750 patients (André-Obadia et al., 2008, 2011, 2018; Ma et al., 2015; Attal et al., 2016, 2021; Hosomi et al., 2013, 2020; Lefaucheur et al., 2006,b; Mori et al., 2022; Quesada et al., 2020; Zhao et al., 2020, 2021). All but one found positive results, and a recent report of the influential US Department of Veterans Affairs using a best-evidence approach concluded that rTMS ‘could be a treatment option for patients who have exhausted other available options for treatment of chronic neuropathic pain’(Anderson et al., 2020). In accordance, rTMS is currently recommended in different guidelines in Europe, Latin America and the USA as a treatment option for chronic pain patients, in particular neuropathic pain (Baptista et al., 2019; Lefaucheur et al., 2014, 2020; Leung et al., 2020; Moisset et al., 2021). Studies on specific parameters of stimulation have helped to design ‘optimized’ rTMS protocols at the group level: Thus, in neuropathic pain (NP) stimulation of the primary motor cortex with 1600–3000 pulses at 10 or 20 Hz is preferred to stimulation at lower frequencies (Mori et al., 2022) and to theta-burst stimulation (André-Obadia et al., 2021; Lefaucheur et al., 2012). Also, a posterior-to-anterior coil orientation was found superior to a latero-medial position (André-Obadia et al., 2008), and M1 superior to DLPFC as a target to relieve NP (Attal et al., 2021). Pain relief after rTMS could be maintained for several months by spaced sessions repeated weekly, fortnightly and even monthly in selected patients with neuropathic pain (Lefaucheur et al., 2004; Kobayashi et al., 2015; Attal et al., 2021) or fibromyalgia (Mhalla et al., 2011; Passard et al., 2007). Overall, after 5 daily induction sessions such ‘optimized’ approaches provided a 25–50% decrease in pain intensity in around half of the treated patients (Baptista et al., 2019; Lefaucheur et al., 2014, 2020) and the benefit could be extended up to 3–6 months using longer inter-sessions periods (Attal et al., 2021; Hodaj et al., 2020; Quesada et al., 2020).

The above advances represent a significant gain, if we consider that patients are addressed to rTMS because of poor response to optimized drug treatment and may therefore obtain an additional improvement in pain control. But these studies also demonstrate something that has been long learned from pharmacological trials, namely the extremely heterogeneous inter-individual response to rTMS effects. Patients develop chronic pain by different pathophysiological backgrounds, whereas any single treatment, be it pharmacological or neuromodulator, has a limited number of mechanisms of action and is, therefore, condemned to work in only a limited number of patients. Any type of monotherapy, whether pharmacologic, neuromodulator, surgical or psychological, is, therefore, bound to yield a proportion of ‘non-responders’, whose mechanisms of disease do not match those of the (mono)therapy (e.g. Bouwense et al., 2015; Holbech et al., 2015). As rTMS slowly enters treatment guidelines for the management of chronic pain, it also starts to be confronted with this limitation. This calls for a collaborative effort to understand how rTMS can be prescribed in a way that matches an individual’s mechanisms of pain, and thus improve treatment efficacy.

**Figure 1:**
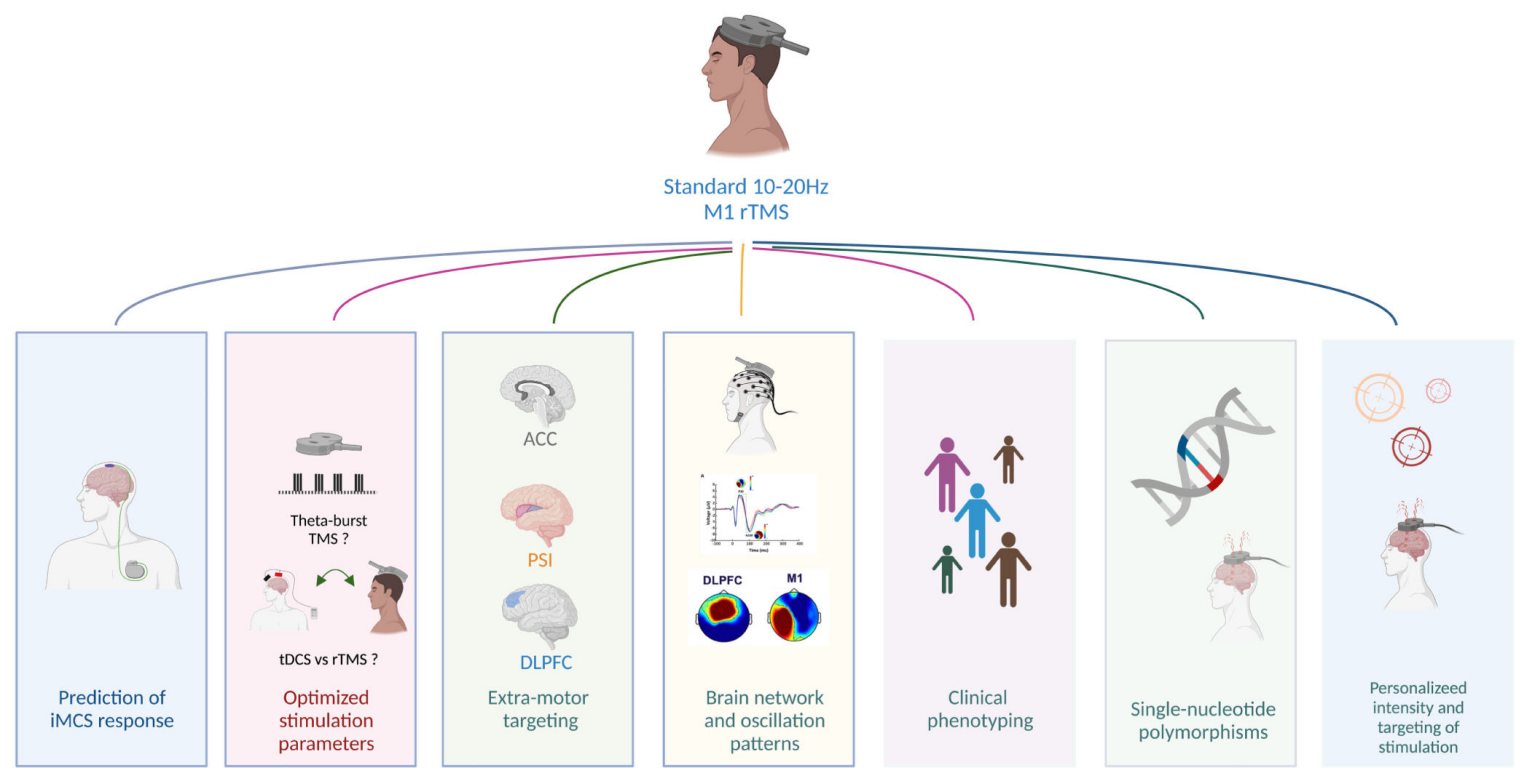
Main strategies to personalize rTMS treatment for neuropathic pain. DLPFC, dorsolateral prefrontal cortex; iMCS, implanted epidural motor cortex stimulation; M1, primary motor cortex; PSI, posterior superior insula; rTMS, repetitive transcranial magnetic stimulation; tDCS, transcranial direct current stimulation. Figure designed with Biorender.

Below we discuss a series of potential paths to overcome this therapeutic challenge in a personalized medicine framework (Figure 1; Table 1).

## 4 Using Rtms to Predict Individual Response to Implanted MCS

Personalized medicine was at the core of the initial studies of rTMS for pain since rTMS was originally used as a screening tool to select patients for surgery, based on the premise that those responding to rTMS would be also likely to respond to implanted stimulation of the same region (iMCS). Cumulative evidence from 7 studies (6 controlled) in 150 operated patients supports the notion that a positive preoperative rTMS can predict satisfactory pain relief after epidural implantation in 80–90% of cases (André-Obadia et al., 2006, 2014; Hosomi et al., 2008; Lefaucheur et al., 2011; Pommier et al., 2018; Saitoh et al., 2006; Zhang et al., 2017). The heterogeneity of patients is however underlined by a recent negative study, where a single preoperative TMS session was unable to predict MCS outcome (Hamani et al., 2021). This may suggest that recurrent, rather than single rTMS sessions should be explored before establishing a prognosis for iMCS (Pommier et al., 2018). Whatever the case, most neurosurgeons would not currently implant patients surgically unless a previous positive rTMS trial has been verified.

## 5 Changing Stimulation Modality Without Changing Brain Targets

### 5.1 Standard versus patterned rTMS modes

Different from standard high-frequency rTMS, ‘theta-burst stimulation’ refers to the delivery of bursts of three consecutive pulses at 50 Hz t (i.e. 20 ms inter-pulse interval), which are repeated at theta frequencies (5 Hz). ‘Theta-burst stimulation’ was initially considered as a potentially advantageous modification of rTMS based on its greater excitability effects on M1 (Huang et al., 2005) and on the results of some experimental studies in healthy subjects (De Martino et al., 2019; Moisset et al., 2015). However, a head-to-head comparison of 46 patients with neuropathic pain showed a significant advantage of standard high-frequency rTMS over the theta-burst procedure, both in terms of pain relief and in the proportion of responding individuals (André-Obadia et al., 2021). Of notice, 25% of patients who did not respond to standard rTMS obtained pain relief from theta bursts, suggesting that this modality may still be proposed in case of failure of standard techniques. Also, the theta-burst procedure could represent a useful means to ‘prime’ the pain-relieving effect of rTMS if it is applied immediately before a standard rTMS session. Thus, in 14 neuropathic pain patients, the average relief passed from 20% to 33% following theta-burst priming, and the proportion of responders to 10 Hz rTMS could be almost tripled when preceded by 600 pulses of intermittent theta burst stimulation (TBS) (Lefaucheur et al., 2012). Although these data still lack replication, they suggest that stimulation paradigms that may not be ‘first line’ can find usefulness in combination, using the same target, coil type and intensity of stimulation. These data also suggest that being a ‘responder’ or ‘non responder’ to TMS may depend on the stimulation paradigm and that refractory patients may become responders by changing the parametric setup. Although identifying good candidates for TBS would clearly improve personalized approaches, none of the multiple clinical criteria analysed so far have allowed for this.

### 5.2 Fields versus currents?

Neuromodulation of cortical neurons using low-intensity direct currents (tDCS) had been described in animal models since the 1960s (Bindman et al., 1964) but did not emerge as a potential therapy for chronic pain until the beginning of this century (Fregni et al., 2006). In a first head-to-head trial, while 10 Hz rTMS to M1 relieved peripheral neuropathic pain due to radiculopathy, anodal tDCS proved not better than sham stimulation (Attal et al., 2016). Two larger and cross-over head-to-head trials, however, showed that both tDCS and rTMS could relieve neuropathic pain to comparable levels, with only a slight superiority of rTMS in the reported amount of pain relief (André-Obadia et al., 2023; Bonifácio de Assis et al., 2022). In these two studies, responders to one technique were not necessarily responders to the other, probably due to the different mechanisms driving pain relief in each of them; the corollary being that both modalities probably deserve to be tested before declaring a patient as unresponsive to cortical neuromodulation (e.g. Hodaj et al., 2020). As it was the case with theta burst stimulation, no predictive markers of response to one or the other approach are available to date. In the absence of therapeutic alternatives for an individual patient, we might suggest systematically giving a chance to both modalities, since tDCS is a less costly technique than rTMS, and can be safely performed outside hospital settings to achieve long-lasting effects (Garcia-Larrea et al., 2019).

## 6 Stimulating Extra-Motor Targets

### 6.1 The prefrontal cortex

The first approved clinical use of rTMS was to control refractory major depression, and the target was the left dorsolateral frontal cortex (O’Reardon et al., 2007). Given the substantial evidence supporting the role of the DLPFC in the cognitive processing and modulation of pain, and the fact that mood symptoms are ominous in patients with chronic pain, the DLPFC was also the first extra-motor target to be tested for pain relief. Initial studies in acute pain models reinforced the idea that this target could have acute analgesic effects similar to M1 stimulation, with different mechanisms of action (Ciampi de Andrade et al., 2014; de Andrade et al., 2011; Nahmias et al., 2009).

Regarding neuropathic pain, initial positive results of DLPFC stimulation in small series (Nardone et al., 2017) failed to be confirmed in larger samples (De Oliveira et al., 2014), even when targeted by neuronavigation (Attal et al., 2021; Mylius et al., 2013). Additionally, DLPFC stimulation did not have an effect on human models of neuropathic hyperalgesia or migraine (Conforto et al., 2014; Sacco et al., 2014). In accordance, recent systematic reviews have considered DLPFC rTMS as either ineffective versus sham (Hamid et al., 2019; O’Connell et al., 2018), less effective than M1 stimulation (Attia et al., 2021) or mildly effective in the short-term (Che et al., 2021).

In non-neuropathic conditions, initial reports of a decrease in postoperative morphine use by DLPFC stimulation (Borckardt et al., 2006) were, however, contradicted by large-scale studies from the same group (Borckardt et al., 2014; Imperatore et al., 2021). Also, while some studies aimed at fibromyalgia were positive in the short term (Cheng et al., 2019; Forogh et al., 2021; Sampson et al., 2006), in most other reports DLPFC stimulation proved to be no better than placebo (Short et al., 2011; Fitzgibbon et al., 2017; Bilir et al., 2021; review in Anderson et al., 2020).

### 6.2 The operculo-insular cortex

The integration and processing of nociceptive inputs in the brain involve multiple cortical areas beyond sensory-motor and prefrontal cortices (Garcia-Larrea & Bastuji, 2018; Kucyi & Davis, 2017; Peyron et al., 2019). Structures of the so-called ‘nociceptive matrix’ such as the parietal operculum and posterior insula are the target of most spinothalamic projections reaching the primate cortex (Dum et al., 2009; Garcia-Larrea & Peyron, 2013), and experimental studies in animals have reported that epidural or deep brain stimulation of insular areas analogous to the human posterior insula led to analgesia, which needed opioid, cannabinoid and/or GABA receptors availability to occur (Alonso-Matielo et al., 2021; Chehade et al., 2021; Dimov et al., 2018; Franca et al., 2013; Komboz et al., 2022). In humans, direct electric stimulation of the posterior superior insula at ‘inhibitory’ high frequencies (150 Hz) increased heat pain thresholds in patients undergoing stereo-EEG for focal epilepsy surgery (Denis et al., 2016). Non-invasive targeting of the posterior insula can be achieved in men with the use of coils allowing for stimulation of deep brain structures, such as the double-angulated coils used to stimulate the distal leg M1 representation buried between the hemispheres (Ciampi de Andrade et al., 2012; da Cunha, Tanaka, et al., 2022). Using this approach, Lenoir et al. (2018) reported a decrease in thermo-nociceptive perception in healthy volunteers when applying deep rTMS to the posterior insula via continuous theta-burst, and similar changes in heat pain thresholds (‘anti-allodynic’ effects) were obtained in patients with central neuropathic pain receiving 10 Hz insular rTMS (Galhardoni et al., 2019). However, while there is emerging consensus that insular stimulation can change the perception of external stimuli, its effect on clinical ongoing pain remains equivocal: no clinical improvement was found in central neuropathic pain patients receiving deep insular rTMS (Galhardoni et al., 2019), while in a subsequent study patients with peripheral neuropathic pain showed analgesic responses to the same mode of stimulation (Dongyang et al., 2021). Although these data need replication, they tend to underscore again that different subsets of patients may be responsive to different types of intervention. If the central/peripheral dissociation were confirmed by further studies, it could imply that insular rTMS is to be preferentially applied in instances where the central nervous system is structurally intact—which in turn would have relevance in the personalized approach to patients with NP.

### 6.3 The anterior cingulate cortex

The anterior cingulate cortex (ACC) is part of a multimodal network implicated in multiple tasks including attentional steering, anticipation and decision-making. It also serves as part of a platform where input from visceromotor areas is confronted with actual somatosensory information, allowing for prediction error estimation and optimal allostasis (Friston, 2010; Owens et al., 2018; Seth & Friston, 2016). In the past century, neurosurgical ACC deafferentation procedures (cingulotomy) were shown to modify the cognitive and affective appraisal of pain, rather than its intensity (Foltz & White, 1962) but were discontinued because of their morbidity. In the same line, optogenetic stimulation of ACC in rats submitted to experimental models of neuropathic pain led to anxiolytic effects without affecting lesion-induced hypersensitivity (Barthas et al., 2015). In healthy human volunteers, non-invasive rTMS aimed at the ACC reduced experimental pain when applied at low frequencies considered as ‘inhibitory’ (1 Hz) (Tzabazis et al., 2013), and neurosurgical ACC stimulation at very high rates of 150 Hz (also considered inhibitory) was also reported to alleviate sensory and affective components of neuropathic pain in a small series of 12 patients (Boccard et al., 2017). In a longer series of central neuropathic pain (*n*=33), ACC rTMS at 10 Hz had a marked positive effect on pain-related anxiety, while the clinical pain intensity was not modified (Galhardoni et al., 2019). Despite the incertitude on frequencies and exact mode of stimulation, these reports suggest that ACC rTMS could be potentially useful in patients not responding to M1 stimulation. The limited amount of data available, the uncertainty regarding the reliability of anatomical targeting and the huge disparity in pain types and stimulation modalities prevent definitive conclusions from being drawn at this stage. The possibility that ACC modulation may improve affective symptoms such as anxiety or depression linked to chronic pain is very attractive; however, the potential adverse effects on cognition and mood from chronic perturbation of ACC function (personality changes, apathy, deficits in self-initiated behaviour) need to be further explored (Cohen et al., 1999, 2001; Hunt et al., 2019). Again, we are confronted with potentially useful techniques that will only be reasonably used when a personalized approach based on consensual phenotyping becomes available.

## 7 Networks and Rhythms to Personalize Treatment?

Neuroscience is increasingly using concepts from network dynamics to understand how brain areas engage to create perception, behaviour, and consciousness (Bassett & Sporns, 2017; Casali et al., 2013; Sporns, 2013). Network science has provided a novel way to look at the modular organization of the brain and to probe its complexity, which has been so far seldom applied to neuromodulation. Networks are composed of nodes (neuronal groups) that communicate with each other by links (axonal projections). Brain networks have localized clusters of activity that can convey local information to distant regions via long-range connections including trans-thalamic routes (Bastuji et al., 2023; Sampathkumar et al 2021). Connectivity patterns between brain areas and networks during neurostimulation have been explored by coupling TMS with MRI and high-density EEG measurements. TMS-EEG allows assessing how stimulating one brain area interferes with activity in distant cortical regions, and performs a mapping of connectivity strength between parts of different networks in health and disease (Cury et al., 2020; Mueller et al., 2018; Rădulescu et al., 2021; Rantamäki & Yalcin, 2016).

Although inter-areal activity binding can also be measured by correlated changes in MRI-recorded oxygen consumption in different regions, the temporal resolution of fMRI is much too slow to guide neurostimulation procedures. Accumulating evidence suggests that brain networks communicate via oscillatory activity at different frequencies, and a potential strategy to improve rTMS therapy is to assess individually such oscillatory activity to tailor treatments (Friston, 2011; Hallett et al., 2020; Tremblay et al., 2019). Synchronized synaptic activity produced by cortical neurons can be recorded in the form of electroencephalogram (EEG) oscillations (Pfurtscheller & Lopes Da Silva, 1999), which correspond well to the range of frequencies used in cortical neuromodulation. Neuronal oscillations link and unify network activity and create moments in time when depolarization of neuronal groups is more or less likely to occur (Buzsáki, 2010). This selectivity allows information to be transferred to distant brain areas and hence influences global brain activity (Buzsáki & Draguhn, 2004; Casali et al., 2009; Lisman & Buzsáki, 2008). Sensorimotor networks oscillate spontaneously at 10–20Hz, (Feurra et al., 2011; Niedermeyer, 1999; Salmelin et al., 1995), which are precisely the frequencies that have been found most useful for pain relief when stimulating the motor cortex (review Garcia-Larrea & Quesada, 2022). This is in accord with Hebbian models (Scarpetta et al., 2002), which postulate an increase in synaptic efficacy when phase locking between the driving stimulus (rTMS) and the intrinsic network frequency is high, as this strengthens the impact of the synchronously firing neurons onto common targets. Analogous to other neuropsychiatric conditions, chronic neuropathic pain has been associated with abnormal oscillatory activity in specific EEG bands, notably increases in amplitude and coherence of theta rhythms at 4–7 Hz (Llinás et al., 1988; Sarnthein & Jeanmonod, 2008) and slowing of frequency oscillations in the alpha band (8–12 Hz) (Kisler et al., 2020; Mussigmann et al., 2022). It remains unsettled whether these abnormalities are directly related to the pain state or rather the result of medication for neuropathic pain, which can induce similar EEG changes (Blume, 2006). Notwithstanding this important confounding factor, applying rTMS at frequencies that are physiological for a given region may be beneficial to restore oscillatory activity to physiological range, and hence contribute to its therapeutic effects (Okazaki et al., 2021; Rosanova et al., 2009). Indeed, ‘optimal’ M1 rTMS is already performed within the 10–20 Hz range (see above), and most studies using these frequencies were generally positive relative to modalities using different frequencies or stimulation patterns (André-Obadia et al., 2021; Mori et al., 2022).

Founded on the results from M1 stimulation, most rTMS studies for pain relief aiming at non-motor targets also used 10–20 Hz frequencies. This appears counter-intuitive since targets such as the ACC, PSI and DLPFC could better be modulated by stimulation frequencies adapted to their natural frequencies (Rosanova et al., 2009). While extensive literature has explored the frequency of oscillatory activities in the prefrontal cortex, the peak frequency of deeper cortical structures such a the insula and cingulate has not been completely described and would be challenging to determine it using surface EEG. The neurophysiological exploration of these targets using intracerebral EEG performed during the pre-operative screening of focal epilepsy could in the near future contribute to a better knowledge of the baseline EEG peak frequencies in health and disease (Gélébart et al., 2023; Liberati et al., 2019).

Besides oscillatory frequency, the phase (negative or positive) of the oscillatory EEG precentral rhythm can also heavily influence the size of the motor response to a TMS pulse, since for instance pulses arriving during the negative-going deflections on the M1-EEG are associated with larger MEPs, (Desideri et al., 2019; Schaworonkow et al., 2019; Zrenner et al., 2018). It is, therefore, tempting to envisage rTMS systems that would be selectively triggered by one of the phases of the oscillatory EEG, depending on whether we desire activating or inhibiting effects on the underlying cortex. While it is unknown whether current EEG systems would allow for such real-time close-loop stimulation strategies in the 10–20 Hz frequency bands, such an approach was recently shown to be feasible at lower frequency ranges (theta) on the DLPFC for the control of major depression (Gordon et al., 2021).

## 8 Genotyping to Select Candidates?

Single nucleotide polymorphism (SNP) refers to the variation of a single base pair in the genome between one individual and the reference ‘standard’ sequence of the species. These variations are very common (approximately one in every thousand base pairs in the human genome), and account for 90% of all human genetic variation. SNPs underlie differences in susceptibility to many diseases, including some inducing pain, and can modify pain thresholds especially when pain modulatory pathways are challenged (Vetterlein et al., 2023); it is, therefore, conceivable that they may contribute to the heterogeneity of response to rTMS. Since monoaminergic mechanisms modulate pain responses, and polymorphisms in dopamine-related genes can change functional connectivity following rTMS (Hong et al., 2023), a number of authors assessed the influence of the dopamine (DRD2) and COMT gene polymorphisms on the rTMS analgesic effect. The homozygous DRD2 T/T genotype was found associated with a positive response to M1 rTMS in two studies gathering patients with orofacial (*n* = 16) or central post-stroke pain (*n* = 17; Jääskeläinen et al., 2014; Ojala et al., 2022). Conversely, no correlations were found between the rTMS outcome and polymorphisms in either DRD2 or COMT Val/Met genotypes, in patients with orofacial or central post-stroke pain stimulated over M1 or S2 (Lindholm et al., 2015; Ojala et al., 2022). The impact on cortical modulation of SNPs of the Brain-Derived-Neurotrophic-Factor (BDNF) gene has been abundantly investigated; however, the results are partly contradictory and dependent on the methods used (Chaieb et al., 2014). The Val66Met BDNF SNP was found to be correlated with resting motor threshold in healthy subjects but did not affect other measures of cortical excitability (Saghazadeh et al., 2016). Also, while rTMS was reported to enhance serum levels of BDNF and cortical excitability (Zhao et al., 2020, 2021), this may not be related to genetic variants, since very similar facilitation of cortical excitability by rTMS was found regardless of BDNF Val/Met polymorphisms in healthy subjects and stroke patients (Hwang et al., 2015; Uhm et al., 2015). All in all, only a small number of SNPs have been associated so far with the response to rTMS treatment for pain, and this in very small samples and with partially inconsistent results. There is a disproportion between the huge samples needed to assess genetic variations reliably, and the actual number of subjects included in rTMS protocols; it is therefore anticipated that a long time will elapse before meta-analyses of studies with comparable methodology may be able to indicate reliably whether genotypic screening is a valuable method to select rTMS candidates.

## 9 Could it Be Simpler Than it Seems?—A Look at Patient’s Symptoms

The development of strategies to classify patients with chronic pain according to sensory profiles, imaging, serum biomarkers or psychological traits that may relate to prognosis or treatment is an active field of research. Initial studies failed to find differential effects of rTMS on specific subtypes of neuropathic pain (continuous, evoked, paroxysmal), since pain sensations were modified whatever their type, suggesting an effect on the global pain appraisal rather than on sensory components (André-Obadia et al., 2008). A different approach consists in using questionnaire-based sensory profiles to classify patients into ‘sensory clusters’ (Freeman et al., 2014). This strategy showed, for example, that patients responding positively to botulinum toxin clustered into specific sensory phenotypes and not in others (Bouhassira et al., 2021). This same strategy was recently applied to patients receiving rTMS to the posterior insula and disclosed that none of the responders to the intervention had a sensory phenotype characterized by predominant allodynic symptoms (Dongyang et al., 2021; Cunha & de Andrade 2022). Despite the obvious limitations of such post hoc analyses, the results suggest that selecting specific clinical presentations based on symptom profiles may increase the efficacy of rTMS at the individual level. This highly attractive approach needs, however, prospective validation in large series of patients to avoid the danger of ‘self-fulfilling prophecies’, whereby expectations, correct or incorrect, bring about the expected outcome (Mertens et al., 2022). Indeed, excluding a priori particular pain phenotypes as supposedly refractory to rTMS, instead of including them in stratified randomization, may lead to the impossibility of adequately testing the intervention in all possible profiles of patients.

## 10 Personalizing Location and Intensity of Rtms

While the motor cortex has proved so far, the most effective rTMS target for neuropathic pain relief, the identification of its precise motor spot has been performed in different manners. An anatomical approach was used in studies using dedicated TMS-neuronavigation systems, while in other cases a functional determination of the ‘hot spot’ (the point yielding highest motor responses) was performed not taking anatomical landmarks into consideration. The superiority of one technique over the other is still open to debate. There is evidence that the body representation in the primary motor cortex can change due to deafferentation-related plasticity (Bramati et al., 2019; Karl et al., 2001). From this standpoint, Nurmikko et al. (2016) compared rTMS delivered to the standard ‘hot spot’ of the hand M1 representation versus an alternative location reflecting plastic M1 reorganization. Responder rates were similar for both sites irrespective of the targeting method and independent of pain location. Since best responders to each site were not the same, using both sites for treatment improved the analgesic results overall. Another source of central plastic modification is the treatment itself (i.e. rTMS), and it has been suggested that repetitive series of rTMS can per se introduce plastic changes of the cortical representation of body regions in M1 irrespective of its clinical effects (Bashir et al., 2011; Butler & Wolf, 2007; Lee et al., 2003). It is also conceivable that pain relief itself may represent another potential source of variability in target excitability (Lefaucheur et al. 2006). Plastic changes induced by the effects of rTMS and pain improvement may be related to each other or not, and may both potentially impact the location of the ‘ideal’ cortical M1 area to be targeted. Hence, a ‘follow the hot spot’ strategy, where the stimulation target is determined repetitively before every therapeutic session, could provide different results than targeting iteratively the same hotspot determined at baseline. Although this strategy is being followed by several groups, the magnitude of its effects is unknown and is probably small. Indeed, it has been shown that matching the stimulation site to the somatotopic M1 representation of the painful area did not improve efficacy in neuropathic pain (Andre-Obadia et al., 2018; Jetté et al., 2013; Nurmikko et al., 2016;) or showed very tiny differences after a single-stimulation sham-controlled session (Ayache et al., 2016). In this same vein, intra-areal variability in the precise anatomical targeting within the posterior insula was unrelated to stimulation efficacy (da Cunha, Dongyang, et al., 2022).

Changes in motor threshold during therapy might also influence rTMS efficacy. The rest motor threshold is defined as the minimal stimulation intensity that triggers a small (50 microvolts) motor-evoked response in half of the trials (Rossini et al., 2015). While rest motor threshold remains the fundamental factor to individually determine therapeutic rTMS intensity (Cueva et al., 2016; ter Braack et al., 2019), data from major depression trials suggest that it may significantly change across therapeutic rTMS sessions (Cotovio et al., 2021). This may have practical implications because if motor threshold changes are not assessed before each stimulation session, the intensity of stimulation will be solely based on its pre-treatment determination, with a risk of under or over-treating patients (Zarkowski et al., 2009). Assessing motor thresholds systematically before each stimulation session could be a straightforward and easy way to individualize the dosage of treatment for each patient. This may have important practical consequences, since motor threshold changes during stimulation predicted the treatment response to rTMS in depressive patients (Pretalli et al., 2012), and were recently associated with successful analgesia in neuropathic pain (Zhao et al., 2020, 2021). Should this be confirmed in future studies, tracking changes in motor thresholds during treatment would allow clinicians to pursue a treatment even if clinical signs of improvement have not yet appeared when motor threshold markers suggest the patient is likely to respond to the intervention.

## 11 Conclusions

Personalized and individual treatment strategies for chronic pain management should remain a major objective in both pharmacological and non-pharmacological approaches. Non-invasive stimulation by rTMS currently suffers from the same limitation as pharmacological treatments: while it provides additional relief in a proportion of patients with drug-resistant chronic pain, it leaves 30–50% of them irresponsive to this strategy. We have summarized several approaches to select potential responders and individualize treatment, which are currently being explored in both translational studies and clinical trials. These strategies range from sophisticated attempts to classify patients according to mechanism-symptom paradigms and cortical connectivity, to the investigation of different anatomical targets or the use of sensory and pain phenotypes to identify potential responders. Save rare exceptions coming from the genetic and oncology fields, the evidence-based individualization of treatment is still a largely unachieved task. Personalization of treatment is still done in the classic medical tradition manner: supported by the best scientific evidence available, guided by clinical judgement and pondered by the patient’s personal preferences and values.

## Figures and Tables

**Table 1:** Main strategies to improve personalization of non-invasive cortical neuromodulation for chronic pain relief, with an associated level of evidence.

Procedure	Rationale	Results and predicting factors
Drug challenge to select patients for motor cortex rTMS or iMCS	Test drugs with different mechanisms to select candidates for stimulation	Pain-relieving response to ketamine may increase the chance of response to neurostimulation **(D)**
rTMS to select patients for implanted (epidural) motor cortex stimulation	Response to non-invasive stimulation may help to select the best candidates	A pain-relieving response to rTMS increases the probability of subsequent iMCS efficacy **(B)**
Switching to and from tDCS and rTMS in case of non-response to the first trial	Responders to tDCS and rTMS are not necessarily the same, even when the target (M1) is kept constant	Different individual responders to each technique **(C)**
Somatotopic matching between pain location and M1 stimulation	Pain site-Ml motor representation of pain site matching is important for epidural MCS; unknown if matching is important for rTMS	Somatotopic matching does *NOT* improve rTMS efficacy (B)
Patterned stimulation (theta-burst) if classic rTMS fails	Greater excitability effects on M1 with theta-burst than classic 10–20 Hz rTMS	Classic 10–20 Hz TMS is superior to theta-burst TMS at the group level (B), but responders are not the same in individual basis. Priming classic rTMS with pre-stimulation theta burst TMS could improve efficacy relative to rTMS alone **(D)**
Posterior insular stimulation	Main cortical site of projection of nociceptive afferents, analgesic effects in rats and anti-allodynic effects in healthy volunteers when stimulated	Possible analgesic effect for peripheral neuropathic pain **(C)**. but not in central pain (
DLPFC stimulation	Prefrontal cortex is involved in cognitive aspects of pain	DLPFC stimulation does not improve neuropathic pain at the group level **(B)**
Anterior cingulate stimulation	ACC involved in attentional & emotional pain processing	rTMS to ACC has a positive effect on anxiety, but pain intensity is not modified compared to sham stimulation **(C)**
Use of closed-loop procedures to trigger rTMS pulses, use of data from cortical connectivity to personalize treatment	Read-outs of continuous EEG could indicate discrete time intervals where stimulation could have stronger effects. The state of cortical connectivity could indicate the best target to be stimulated on an individual basis (targets less functionally connected than expected would be the ideal stimulation site to improve network performance towards normal	Untested
Sensory phenotyping	Sensory symptoms classified in clusters have been reported to predict response to a number of drugs	Non-responders to insular rTMS may be predominant of the ‘evoked pain’ NPSI phenotype **(D)**
Assessment of hot spot location, rest motor threshold across session and correction of stimulation settings accordingly	Changes in cortical M1 representation of a pain area and its rest motor threshold could be affected by a secondary disease (stroke), by pain itself, by pain medication and by pain improvement. Adjustments of these parameters across sessions would allow thus prevent over or under-stimulation of the target area on M1	Untested
Genetic polymorphisms	Certain polymorphisms in COMT, dopamine and BDNF-related genes can influence pain sensitivity and could affect the response to rTMS	Specific polymorphisms of the dopamine 2 receptor (DRD_2_ T/T) in patients with orofacial and post-stroke pain **(D)**. Not yet proof of effect of specific COMT and BDNF variants **(D)**

*Note:* rTMS (repetitive transcranial magnetic stimulation), iMCS (implanted motor cortex stimulation), DLPFC (dorsolateral prefrontal cortex), M1 (primary motor cortex), ACC (anterior cingulate cortex), PSI (posterior superior insula), BDNF (brain-derived neurotrophic factor), D: dopamine, COMT(catechol-O-methyltransferase). Levels of confidence level to the risk of bias (adapted from AAN (Gronseth et al., 2017) and Lefaucheur et al. (2020)). *Evidence Level A* (‘high’): At least 2 convincing Class I studies from independent teams, OR one Class I and 2 Class II study. *Evidence Level B* (‘moderate’): 1 Class I OR at least 2 convincing Class II studies, *Evidence Level C* (‘low’): One convincing Class II study alone, OR at least 2 convincing Class III *studies.Evidence level D* (‘very low’): Absence of at least 2 convincing Class III studies. *Evidence level U*: (*‘unknown’*): Ever yet tested.

## Study classes

*A Class I study* is an adequately data-supported, prospective, randomized, placebo-controlled clinical trial with masked outcome assessment in *n* ≥ 25 patients receiving active treatment. It should include (a) randomization concealment; (b) clearly defined primary outcomes; (c) clearly defined exclusion/inclusion criteria; (d) adequate accounting for dropouts and crossovers with numbers sufficiently low to have minimal potential for bias and (e) relevant baseline characteristics substantially equivalent among treatment groups or appropriate statistical adjustment for differences.

*A Class II study* is a randomized, placebo-controlled trial performed with a smaller sample size (*n* < 25) or that lacks at least one of the above-listed criteria.

*Class III studies* include all other controlled trials. (Including well-defined external, natural history controls) in a representative population, where the outcome is independently assessed, or independently derived by objective outcome measurement.

*Class IV studies* are uncontrolled studies, case series and case reports.

## Panel 1: Case vignette

A 48-year-old woman presented with neuropathic pain (definite NP according to the IASP classification) 1 year after a traumatic lesion to the radial nerve. The pain was localized to the left arm and did not respond to first and second-line treatments, including lidocaine patches, intra-cutaneous botulinum toxin injections (2 trials) and gabapentin. Small doses of tricyclic antidepressant (amitriptyline 12.5 mg qd.) decreased pain intensity but caused intolerable constipation and sleepiness. Duloxetine 90 mg qd. was used with a 40% pain intensity reduction, which was not enough to improve quality of life. She maintained major functional impairment, with pain affecting mood, sleep and work performance. High-frequency repetitive transcranial magnetic stimulation (rTMS) was suggested as a third line of treatment, and was added to duloxetine, physical rehabilitation and psychological care. After safety screening clearance and realistic discussion about expected results, rTMS to the hand representation on the right primary motor cortex was performed (figure-of-eight coil, biphasic pulses with the main phase in the antero-posterior direction). She underwent five daily sessions of 3000 pulses each when she started to perceive some pain improvement. The treatment was then delivered weekly for 7 weeks, and then fortnightly for 3 months. Mild and short-lasting headaches were felt after the first sessions, were managed with paracetamol and then disappeared. During treatment, the measurement of the rest motor threshold showed a steady decrease up to 15% below the pretreatment value, and for that reason, the intensity of stimulation was adjusted based on its value before each session. Pain intensity decreased by a further 40% while medication and rehabilitation treatment were maintained during the whole period.

Due to a travel during summer vacations, the treatment was discontinued for a month and pain intensity increased after 3 weeks without rTMS. Upon return home, a new run of induction and maintenance sessions allowed the clinical improvement to be restored. The patient is centred on long-term autonomy and independence from hospital visits for treatment. She is currently discussing with the healthcare team the possibility to maintain stimulations at home with transcranial direct current stimulation (anodal to M1) or to undergo implanted motor cortex stimulation. She is currently inclined to try the former first.

## References

[R1] Ahmed MA, Mohamed SA, Sayed D (2011). Long-term antalgic effects of repetitive transcranial magnetic stimulation of motor cortex and serum beta-endorphin in patients with phantom pain. Neurological Research.

[R2] Alonso-Matielo H, Goncalves ES, Campos M, Oliveira VRS, Toniolo EF, Alves AS, Lebrun I, de Andrade DC, Teixeira MJ, Britto LRG, Hamani C (2021). Electrical stimulation of the posterior insula induces mechanical analgesia in a rodent model of neuropathic pain by modulating GABAergic signaling and activity in the pain circuitry. Brain Research.

[R3] Anderson J, Parr NJ, Vela K (2020). Evidence Brief: Transcranial Magnetic Stimulation (TMS) for Chronic Pain, PTSD, TBI, Opioid Addiction, and Sexual Trauma. Evidence Brief: Transcranial Magnetic Stimulation (TMS) for Chronic Pain, PTSD, TBI, Opioid Addiction, and Sexual Trauma.

[R4] André-Obadia N, Hodaj H, Hodaj E, Simon E, Delon-Martin C, Garcia-Larrea L (2023). Better fields or currents? A head-to-head comparison of transcranial magnetic (rTMS) versus direct current stimulation (tDCS) for neuropathic pain. Neurotherapeutics.

[R5] André-Obadia N, Magnin M, Garcia-Larrea L (2011). On the importance of placebo timing in rTMS studies for pain relief. Pain.

[R6] André-Obadia N, Magnin M, Garcia-Larrea L (2021). Thetaburst versus 20 Hz repetitive transcranial magnetic stimulation in neuropathic pain: A head-to-head comparison. Clinical Neurophysiology.

[R7] Andre-Obadia N, Magnin M, Simon E, Garcia-Larrea L (2018). Somatotopic effects of rTMS in neuropathic pain? A comparison between stimulation over hand and face motor areas. European Journal of Pain (United Kingdom).

[R8] André-Obadia N, Mertens P, Gueguen A, Peyron R, Garcia-Larrea L (2008). Pain relief by rTMS: Differential effect of current flow but no specific action on pain subtypes. Neurology.

[R9] André-Obadia N, Mertens P, Lelekov-Boissard T, Afif A, Magnin M, Garcia-Larrea L (2014). Prospective evaluation is life better after motor cortex stimulation for pain control? Results at long-term and their prediction by preoperative rTMS. Pain Physician.

[R10] André-Obadia N, Peyron R, Mertens P, Mauguière F, Laurent B, Garcia-Larrea L (2006). Transcranial magnetic stimulation for pain control. Double-blind study of different frequencies against placebo, and correlation with motor cortex stimulation efficacy. Clinical Neurophysiology: Official Journal of the International Federation ofClinical Neurophysiology.

[R11] Attal N, Ayache SS, Ciampi De Andrade D, Mhalla A, Baudic S, Jazat F, Ahdab R, Neves DO, Sorel M, Lefaucheur JP, Bouhassira D (2016). Repetitive transcranial magnetic stimulation and transcranial direct-current stimulation in neuropathic pain due to radiculopathy: A randomized sham-controlled comparative study. Pain.

[R12] Attal N, Poindessous-Jazat F, De Chauvigny E, Quesada C, Mhalla A, Ayache SS, Fermanian C, Nizard J, Peyron R, Lefaucheur JP, Bouhassira D (2021). Repetitive transcranial magnetic stimulation for neuropathic pain: A randomized multicentre sham-controlled trial. Brain: A Journal of Neurology.

[R13] Attia M, McCarthy D, Abdelghani M (2021). Repetitive tran-scranial magnetic stimulation for treating chronic neuropathic pain: A systematic review. Current Pain and Headache Reports.

[R14] Ayache SS, Ahdab R, Chalah MA, Farhat WH, Mylius V, Goujon C (2016). Analgesic effects of navigated motor cortex rTMS in patients with chronic neuropathic pain. European Journal of Pain.

[R15] Baptista AF, Fernandes AMBL, Sa KN, Okano AH, Brunoni AR, Lara-Solares A, Jreige Iskandar A, Guerrero C, Amescua-Garcia C, Kraychete DC, Caparelli-Daquer E (2019). Latin American and Caribbean consensus on noninvasive central nervous system neuromodulation for chronic pain management (LAC2-NIN-CP). Pain Reports.

[R16] Barker AT, Jalinous R, Freeston IL (1985). Non-invasive magnetic stimulation of human motor cortex. Lancet (London, England).

[R17] Barthas F, Sellmeijer J, Hugel S, Waltisperger E, Barrot M, Yalcin I (2015). The anterior cingulate cortex is a critical hub for pain-induced depression. Biological Psychiatry.

[R18] Bashir S, Edwards D, Pascual-Leone A (2011). Neuronavigation increases the physiologic and behavioral effects of low-frequency rTMS of primary motor cortex in healthy subjects. Brain Topography.

[R19] Bassett DS, Sporns O (2017). Network neuroscience. Nature Neuroscience.

[R20] Bastuji H, Cadic-Melchior A, Glaunec LR, Magnin M, Garcia-Larrea L (2023). Functional connectivity between medial pulvinar and cortical networks as a predictor of arousal to noxious stimuli during sleep. The European Journal of Neuroscience.

[R21] Bestmann S, Baudewig J, Siebner HR, Rothwell JC, Frahm J (2004). Functional MRI of the immediate impact of transcranial magnetic stimulation on cortical and subcortical motor circuits. The European Journal of Neuroscience.

[R22] Bickford RG, Guidi M, Fortesque P, Swenson M (1987). Magnetic stimulation of human peripheral nerve and brain. Neurosurgery.

[R23] Bilir I, Askin A, Sengul I, Tosun A (2021). Effects of high-frequency Neuronavigated repetitive transcranial magnetic stimulation in fibromyalgia syndrome: A double-blinded, randomized controlled study. American Journal of Physical Medicine & Rehabilitation.

[R24] Bindman LJ, Lippold OCJ, Redfearn JWT (1964). The action of brief polarizing currents on the cerebral cortex of the rat (1) during current flow and (2) in the production of long-lasting after-effects. The Journal of Physiology.

[R25] Blume WT (2006). Drug effects on EEG. Journal of Clinical Neurophysiology.

[R26] Boccard SGJ, Prangnell SJ, Pycroft L, Cheeran B, Moir L, Pereira EAC, Fitzgerald JJ, Green AL, Aziz TZ (2017). Long-term results of deep brain stimulation of the anterior cingulate cortex for neuropathic pain. World Neurosurgery.

[R27] Bonifácio de Assis ED, Martins WKN, de Carvalho CD, Ferreira CM, Gomes R, de Almeida Rodrigues ET, Meira UM, de Holanda LJ, Lindquist AR, Morya E, Mendes CKTT (2022). Effects of rTMS and tDCS on neuropathic pain after brachial plexus injury: A randomized placebo-controlled pilot study. Scientific Reports.

[R28] Borckardt JJ, Reeves ST, Kotlowski P, Abernathy JH, Field LC, Dong L, Frohman H, Moore H, Ryan K, Madan A, George MS (2014). Fast left prefrontal rTMS reduces post-gastric bypass surgery pain: Findings from a large-scale, double-blind, sham-controlled clinical trial. Brain Stimulation.

[R29] Borckardt JJ, Weinstein M, Reeves ST, Kozel FA, Nahas Z, Smith AR, Karl Byrne T, Morgan K, George MS (2006). Postoperative left prefrontal repetitive transcranial magnetic stimulation reduces patient-controlled analgesia use. Anesthesiology.

[R30] Bouhassira D, Branders S, Attal N, Fernandes AM, Demolle D, Barbour J, Ciampi de Andrade D, Pereira A (2021). Stratification of patients based on the neuropathic pain symptom inventory: Development and validation of a new algorithm. Pain.

[R31] Bouwense SAW, Olesen SS, Drewes AM, van Goor H, Wilder-Smith OHG (2015). Pregabalin and placebo responders show different effects on central pain processing in chronic pancreatitis patients. Journal of Pain Research.

[R32] Bramati IE, Rodrigues EC, Simões EL, Melo B, Höfle S, Moll J, Lent R, Tovar-Moll F (2019). Lower limb amputees undergo long-distance plasticity in sensorimotor functional connectivity. Scientific Reports.

[R33] Buzsáki G (2010). Neural syntax: Cell assemblies, synapsembles, and readers. Neuron.

[R34] Butler AJ, Wolf SL (2007). Putting the brain on the map: use of transcranial magnetic stimulation to assess and induce cortical plasticity of upper-extremity movement. Physical Therapy.

[R35] Buzsáki G, Draguhn A (2004). Neuronal olscillations in cortical networks. Science.

[R36] Casali AG, Casarotto S, Rosanova M, Mariotti M, Massimini M (2009). General indices to characterize the electrical response of the cerebral cortex to TMS. NeuroImage.

[R37] Casali AG, Gosseries O, Rosanova M, Boly M, Sarasso S, Casali KR, Casarotto S, Bruno MA, Laureys S, Tononi G, Massimini M (2013). A theoretically based index of consciousness independent of sensory processing and behavior. Science Translational Medicine.

[R38] CFM. Resolution (2012). Resolução CFM 1986/2012.

[R39] Chaieb L, Antal A, Ambrus GG, Paulus W (2014). Brain-derived neurotrophic factor: Its impact upon neuroplasticity and neuroplasticity inducing transcranial brain stimulation protocols. Neurogenetics.

[R40] Che X, Cash RFH, Luo X, Luo H, Lu X, Xu F, Zang YF, Fitzgerald PB, Fitzgibbon BM (2021). High-frequency rTMS over the dorsolateral prefrontal cortex on chronic and provoked pain: A systematic review and meta-analysis. Brain Stimulation.

[R41] Cheng CM, Wang SJ, Su TP, Chen MH, Hsieh JC, Ho ST, Bai YM, Kao NT, Chang WH, Li CT (2019). Analgesic effects of repetitive transcranial magnetic stimulation on modified 2010 criteria-diagnosed fibromyalgia: Pilot study. Psychiatry and clinical neurosciences.

[R42] Chehade HD, Kobaïter-Maarrawi S, Komboz F, Farhat JP, Magnin M, Garcia-Larrea L, Maarrawi J (2021). Somatosensory thalamic activity modulation by posterior insular stimulation: Cues to clinical application based on comparison of frequencies in a cat model. Neuromodulation.

[R43] Ciampi de Andrade D, Galhardoni R, Pinto LF, Lancelotti R, Rosi JJ, Marcolin MA, Teixeira MJ (2012). Into the Island: A new technique of non-invasive cortical stimulation of the insula. Neurophysiologie Clinique = Clinical Neurophysiology.

[R44] Ciampi De Andrade D, Mhalla A, Adam F, Texeira MJ, Bouhassira D (2014). Repetitive transcranial magnetic stimulation induced analgesia depends on N-methyl-d-aspartate glutamate receptors. Pain.

[R45] Cohen RA, Kaplan RF, Zuffante P, Moser DJ, Jenkins MA, Salloway S, Wilkinson H (1999). Alteration of intention and self-initiated action associated with bilateral anterior cingulotomy. The Journal of Neuropsychiatry and Clinical Neurosciences.

[R46] Cohen RA, Paul R, Zawacki TM, Moser DJ, Sweet L, Wilkinson H (2001). Emotional and personality changes following cingulotomy. Emotion.

[R47] Conforto AB, Amaro E, Gonçalves AL, Mercante JPP, Guendler VZ, Ferreira JR, Kirschner CCFB, Peres MFP (2014). Randomized, proof-of-principle clinical trial of active transcranial magnetic stimulation in chronic migraine. Cephalalgia.

[R48] Cotovio G, Oliveira-Maia AJ, Paul C, Faro Viana F, Rodrigues da Silva D, Seybert C, Stern AP, Pascual-Leone A, Press DZ (2021). Day-to-day variability in motor threshold during rTMS treatment for depression: Clinical implications. Brain Stimulation.

[R49] Cueva AS, Galhardoni R, Cury RG, Parravano DC, Correa G, Araujo H, Cecilio SB, Raicher I, Toledo D, Silva V, Marcolin MA (2016). Normative data of cortical excitability measurements obtained by transcranial magnetic stimulation in healthy subjects. Neurophysiologie Clinique.

[R50] Cury RG, Teixeira MJ, Galhardoni R, Silva V, Iglesio R, Franca C, Arnaut D, Fonoff ET, Barbosa ER, Ciampi de Andrade D (2020). Connectivity patterns of subthalamic stimulation influence pain outcomes in Parkinson’s disease. Frontiers in Neurology.

[R51] da Cunha PHM, Dongyang L, Fernandes AM, Thibes RB, Sato J, Tanaka H, Dale C, da Lapa JDS, de Morais ADS, Soares FHC, da Silva VA (2022). Non-invasive insular stimulation for peripheral neuropathic pain: Influence of target or symptom?. Neurophysiologie Clinique.

[R52] da Cunha PHM, Tanaka H, da Lapa JDS, Dongyang L, Boa Sorte AA, Pereira TMR, Soares FHC, Fernandes AM, Aparecida da Silva V, Graven-Nielsen T, Teixeira MJ (2022). The fast-posterior superior insula (fast-PSI): A neuronavigation-free targeting method for non-invasive neuromodulation. Brain Stimulation.

[R53] de Andrade DC, Mhalla A, Adam F, Texeira MJ, Bouhassira D (2011). Neuropharmacological basis of rTMS-induced analgesia: The role of endogenous opioids. Pain.

[R54] De Martino E, Fernandes AM, Galhardoni R, De Oliveira Souza C, Ciampi De Andrade D, Graven-Nielsen T (2019). Sessions of prolonged continuous theta burst stimulation or high-frequency 10 Hz stimulation to left dorsolateral prefrontal cortex for 3 days decreased pain sensitivity by modulation of the efficacy of conditioned pain modulation. The Journal of Pain.

[R55] De Oliveira RAA, De Andrade DC, Mendonça M, Barros R, Luvisoto T, Myczkowski ML, Marcolin MA, Teixeira MJ (2014). Repetitive transcranial magnetic stimulation of the left premotor/dorsolateral prefrontal cortex does not have analgesic effect on central poststroke pain. Journal of Pain.

[R56] Denis DJ, Marouf R, Rainville P, Bouthillier A, Nguyen DK (2016). Effects of insular stimulation on thermal nociception. European Journal of Pain (United Kingdom).

[R57] Desideri D, Zrenner C, Ziemann U, Belardinelli P (2019). Phase of sensorimotor µ-oscillation modulates cortical responses to transcranial magnetic stimulation of the human motor cortex. Journal of Physiology.

[R58] Dimov LF, Toniolo EF, Alonso-Matielo H, de Andrade DC, Garcia-Larrea L, Ballester G, Teixeira MJ, Dale CS (2018). Electrical stimulation of the insular cortex as a novel target for the relief of refractory pain: An experimental approach in rodents. Behavioural Brain Research.

[R59] Doi W, Sato D, Fukuzako H, Takigawa M (2001). c-Fos expression in rat brain after repetitive transcranial magnetic stimulation. Neuroreport.

[R60] Dongyang L, Fernandes AM, da Cunha PHM, Tibes R, Sato J, Listik C, Dale C, Kubota GT, Galhardoni R, Teixeira MJ, Aparecida da Silva V (2021). Posterior-superior insular deep transcranial magnetic stimulation alleviates peripheral neuropathic pain—A pilot double-blind, randomized cross-over study. Neurophysiologie Clinique.

[R61] Dum RP, Levinthal DJ, Strick PL (2009). The spinothalamic system targets motor and sensory areas in the cerebral cortex of monkeys. Journal of Neuroscience.

[R62] Faraday M, Day P (1999). The philosopher’s tree: a selection of Michael Faraday’s writings 211.

[R63] Fernandes AM, Graven-Nielsen T, De Andrade DC (2022). New updates on transcranial magnetic stimulation in chronic pain. Current Opinion in Supportive and Palliative Care.

[R64] Feurra M, Paulus W, Walsh V, Kanai R (2011). Frequency specific modulation of human somatosensorycortex. Frontiers in Psychology.

[R65] Fitzgibbon BM, de Andrade DC, Schabrun SM (2017). Combined cerebral and peripheral treatments for pain: A commentary on Hazime et al. European Journal of Pain.

[R66] Foltz EL, White LE (1962). Pain “relief” by frontal cingulumotomy. Journal of Neurosurgery.

[R67] Forogh B, Haqiqatshenas H, Ahadi T, Ebadi S, Alishahi V, Sajadi S (2021). Repetitive transcranial magnetic stimulation (rTMS) versus transcranial direct current stimulation (tDCS) in the management of patients with fibromyalgia: A randomized controlled trial. Neurophysiologie clinique = Clinical neurophysiology.

[R68] Foy J (2011). K061053-2 department of health and human services—FDA. Neurostar® TMS systems class III designation.

[R69] Franca NRM, Toniolo EF, Franciosi AC, Alves AS, de Andrade DC, Fonoff ET, Britto LR, Dale CS (2013). Antinociception induced by motor cortex stimulation: Somatotopy of behavioral response and profile of neuronal activation. Behavioural Brain Research.

[R70] Freeman R, Baron R, Bouhassira D, Cabrera J, Emir B (2014). Sensory profiles of patients with neuropathic pain based on the neuropathic pain symptoms and signs. Pain.

[R71] Fregni F, Boggio PS, Lima MC, Ferreira MJL, Wagner T, Rigonatti SP, Castro AW, Souza DR, Riberto M, Freedman SD, Nitsche MA (2006). A sham-controlled, phase II trial of transcranial direct current stimulation for the treatment of central pain in traumatic spinal cord injury. Pain.

[R72] Fregni F, Potvin K, Dasilva D, Wang X, Lenkinski RE, Freedman SD, Pascual-Leone A (2011). Clinical effects and brain metabolic correlates in non-invasive cortical neuromodulation for visceral pain. European Journal of Pain.

[R73] Friston K (2010). The free-energy principle: A unified brain theory?. Nature Reviews Neuroscience.

[R74] Friston KJ (2011). Functional and effective connectivity: A review. Brain Connectivity.

[R75] Galhardoni R, Da Silva VA, García-Larrea L, Dale C, Baptista AF, Barbosa LM, Bahia Menezes LM, De Siqueira SRDT, Valério F, Rosi J (2019). Insular and anterior cingulate cortex deep stimulation for central neuropathic pain disassembling the percept of pain. Neurology.

[R76] Gan Z, Gangadharan V, Liu S, Körber C, Tan LL, Li H, Oswald MJ, Kang J, Martin-Cortecero J, Männich D, Groh A (2022). Layer-specific pain relief pathways originating from primary motor cortex. Science (New York, N Y).

[R77] Garcia-Larrea L, Bastuji H (2018). Pain and consciousness. Progress in Neuro-Psychopharmacology & Biological Psychiatry.

[R78] Garcia-Larrea L, Perchet C, Hagiwara K, André-Obadia N (2019). At-home cortical stimulation for neuropathic pain: A feasibility study with initial clinical results. Neurotherapeutics.

[R79] Garcia-Larrea L, Peyron R (2013). Pain matrices and neuropathic pain matrices: A review. Pain.

[R80] Garcia-Larrea L, Quesada C (2022). Cortical stimulation for chronic pain: From anecdote to evidence. European Journal of Physical and Rehabilitation Medicine.

[R81] Gélébart J, Garcia-Larrea L, Frot M (2023). Amygdala and anterior insula control the passage from nociception to pain. Cerebral Cortex.

[R82] Gordon EM, Chauvin RJ, Van AN, Rajesh A, Nielsen A, Newbold DJ, Lynch CJ, Seider NA, Krimmel SR, Scheidter KM, Monk J (2023). A somato-cognitive action network alternates with effector regions in motor cortex. Nature.

[R83] Gordon PC, Dörre S, Belardinelli P, Stenroos M, Zrenner B, Ziemann U, Zrenner C (2021). Prefrontal theta-phase synchronized brain stimulation with real-time EEG-triggered TMS. Frontiers in Human Neuroscience.

[R84] Gronseth GS, Cox J, Gloss D, Merillat S, Dittman J, Armstrong MJ, Getchius TSD (2017). Clinical Practice Guideline Process Manual.

[R85] Hallett M, de Haan W, Deco G, Dengler R, Di Iorio R, Gallea C, Gerloff C, Grefkes C, Helmich RC, Kringelbach ML, Miraglia F (2020). Human brain connectivity: Clinical applications for clinical neurophysiology. Clinical Neurophysiology.

[R86] Hamani C, Fonoff ET, Parravano DC, Silva VA, Galhardoni R, Monaco B, Navarro J, Yeng LT, Teixeira MJ, de Andrade C, Ciampi de Andrade D (2021). Motor cortex stimulation for chronic neuropathic pain: Results of a doubleblind randomized study. Brain: AJournal of Neurology.

[R87] Hamid P, Malik BH, Hussain ML (2019). Noninvasive transcranial magnetic stimulation (TMS) in chronic refractory pain: A systematic review. Cureus.

[R88] Hausmann A, Weis C, Marksteiner J, Hinterhuber H, Humpel C (2000). Chronic repetitive transcranial magnetic stimulation enhances c-fos in the parietal cortex and hippocampus. Brain Research Molecular Brain Research.

[R89] Hodaj H, Payen JF, Hodaj E, Dumolard A, Maindet C, Cracowski JL, Delon-Martin C, Lefaucheur JP (2020). Long-term treatment of chronic orofacial, pudendal, and central neuropathic limb pain with repetitive transcranial magnetic stimulation of the motor cortex. Clinical Neurophysiology.

[R90] Hodkinson DJ, Bungert A, Bowtell R, Jackson SR, Jung JY (2021). Operculo-insular and anterior cingulate plasticity induced by transcranial magnetic stimulation in the human motor cortex: A dynamic casual modeling study. Journal of Neurophysiology.

[R91] Holbech JV, Bach FW, Finnerup NB, Brøsen K, Jensen TS, Sindrup SH (2015). Imipramine and pregabalin combination for painful polyneuropathy: A randomized controlled trial. Pain.

[R92] Hong H, Kim RY, Song Y, Suh C, Lee H, Lyoo IK, Yoon S, Lim SM, Lee S (2023). Genetic profile for dopamine signaling predicts brain functional reactivity to repetitive transcranial magnetic stimulation. European Archives of Psychiatry and Clinical Neuroscience.

[R93] Hosomi K, Saitoh Y, Kishima H, Oshino S, Hirata M, Tani N, Shimokawa T, Yoshimine T (2008). Electrical stimulation of primary motor cortex within the central sulcus for intractable neuropathic pain. Clinical Neurophysiology: Official Journal of the International Federation of Clinical Neurophysiology.

[R94] Hosomi K, Shimokawa T, Ikoma K, Nakamura Y, Sugiyama K, Ugawa Y, Uozumi T, Yamamoto T, Saitoh Y (2013). Daily repetitive transcranial magnetic stimulation of primary motor cortex for neuropathic pain: A randomized, multicenter, double-blind, crossover, sham-controlled trial. Pain.

[R95] Hosomi K, Sugiyama K, Nakamura Y, Shimokawa T, Oshino S, Goto Y, Mano T, Shimizu T, Yanagisawa T, Saitoh Y (2020). A randomized controlled trial of 5 daily sessions and continuous trial of 4 weekly sessions of repetitive transcranial magnetic stimulation for neuropathic pain. Pain.

[R96] Huang YZ, Edwards MJ, Rounis E, Bhatia KP, Rothwell JC (2005). Theta burst stimulation of the human motor cortex. Neuron.

[R97] Hunt PJ, Karas PJ, Viswanathan A, Sheth SA, Sagher O, Levin E, Pilitsis J (2019). Pain Neurosurgery, Neurosurgery by Example.

[R98] Hwang JM, Kim YH, Yoon KJ, Uhm KE, Chang WH (2015). Different responses to facilitatory rTMS according to BDNF genotype. Clinical Neurophysiology.

[R99] Imperatore JP, McCalley DM, Borckardt JJ, Brady KT, Hanlon CA (2021). Non-invasive brain stimulation as a tool to decrease chronic pain in current opiate users: A parametric evaluation of two promising cortical targets. Drug and Alcohol Dependence.

[R100] Jääskeläinen SK, Lindholm P, Valmunen T, Pesonen U, Taiminen T (2014). Variation in the dopamine D2 receptor gene plays a key role in human pain and its modulation by transcranial magnetic stimulation. Pain.

[R101] Jetté F, Côté I, Meziane HB, Mercier C (2013). Effect of single-session repetitive transcranial magnetic stimulation applied over the hand versus leg motor area on pain after spinal cord injury. Neurorehabilitation and Neural Repair.

[R102] Ji RR, Schlaepfer TE, Aizenman CD, Epstein CM, Qiu D, Huang JC, Rupp F (1998). Repetitive transcranial magnetic stimulation activates specific regions in rat brain. Proceedings of the National Academy of Sciences of the United States of America.

[R103] Kadono Y, Koguchi K, Okada KI, Hosomi K, Hiraishi M, Ueguchi T, Kida I, Shah A, Liu G, Saitoh Y (2021). Repetitive transcranial magnetic stimulation restores altered functional connectivity of central poststroke pain model monkeys. Scientific Reports.

[R104] Kan RLD, Padberg F, Giron CG, Lin TTZ, Zhang BBB, Brunoni AR, Kranz GS (2023). Effects of repetitive transcranial magnetic stimulation of the left dorsolateral prefrontal cortex on symptom domains in neuropsychiatric disorders: A systematic review and cross-diagnostic meta-analysis. The Lancet Psychiatry.

[R105] Karl A, Birbaumer N, Lutzenberger W, Cohen LG, Flor H (2001). Reorganization of motor and somatosensory cortex in upper extremity amputees with phantom limb pain. The Journal of Neuroscience.

[R106] Kennedy NI, Lee WH, Frangou S (2018). Efficacy of noninvasive brain stimulation on the symptom dimensions of schizophrenia: A meta-analysis of randomized controlled trials. European Psychiatry: The Journal of the Association of European Psychiatrists.

[R107] Khedr EM, Kotb H, Kamel NF, Ahmed MA, Sadek R, Rothwell JC (2005). Longlasting antalgic effects of daily sessions of repetitive transcranial magnetic stimulation in central and peripheral neuropathic pain. Journal of neurology, neurosurgery, and psychiatry.

[R108] Kisler LB, Kim JA, Hemington KS, Rogachov A, Cheng JC, Bosma RL, Osborne NR, Dunkley BT, Inman RD, Davis KD (2020). Abnormal alpha band power in the dynamic pain connectome is a marker of chronic pain with a neuropathic component. NeuroImage: Clinical.

[R109] Kobayashi M, Fujimaki T, Mihara B, Ohira T (2015). Repetitive transcranial magnetic stimulation once a week induces sustainable long-term relief of central poststroke pain. Neuromodulation.

[R110] Komboz F, Mehsein Z, Kobaïter-Maarrawi S, Chehade HD, Maarrawi J (2022). Epidural Posterior Insular Stimulation Alleviates Neuropathic Pain Manifestations in Rats With Spared Nerve Injury Through Endogenous Opioid System. Neuromodulation.

[R111] Kucyi A, Davis KD (2017). The neural code for pain: From single-cell electrophysiology to the dynamic pain connec-tome. The Neuroscientist.

[R112] Lamusuo S, Hirvonen J, Lindholm P, Martikainen IK, Hagelberg N, Parkkola R, Taiminen T, Hietala J, Helin S, Virtanen A, Pertovaara A (2017). Neurotransmitters behind pain relief with transcranial magnetic stimulation -positron emission tomography evidence for release of endogenous opioids. European Journal of Pain.

[R113] Lee L, Siebner HR, Rowe JB, Rizzo V, Rothwell JC, Frackowiak RSJ, Friston KJ (2003). Acute remapping within the motor system induced by low-frequency repetitive transcranial magnetic stimulation. The Journal of Neuroscience.

[R114] Lefaucheur JP, Aleman A, Baeken C, Benninger DH, Brunelin J, di Lazzaro V, Filipović SR, Grefkes C, Hasan A, Hummel FC, Jääskeläinen SK (2020). Evidence-based guidelines on the therapeutic use of repetitive transcranial magnetic stimulation (rTMS): An update (2014-2018). Clinical Neurophysiology.

[R115] Lefaucheur JP, André-Obadia N, Antal A, Ayache SS, Baeken C, Benninger DH, Cantello RM, Cincotta M, de Carvalho M, De Ridder D, Devanne H (2014). Evidence-based guidelines on the therapeutic use of repetitive transcranial magnetic stimulation (rTMS). Clinical Neurophysiology.

[R116] Lefaucheur JP, Ayache SS, Sorel M, Farhat WH, Zouari HG, Ciampi De Andrade D, Ahdab R, Ménard-Lefaucheur I, Brugières P, Goujon C (2012). Analgesic effects of repetitive transcranial magnetic stimulation of the motor cortex in neuropathic pain: Influence of theta burst stimulation priming. European Journal of Pain.

[R117] Lefaucheur J-P, Drouot X, Keravel Y, Nguyen J-P (2001). Pain relief induced by repetitive transcranial magnetic stimulation of precentral cortex. Neuroreport.

[R118] Lefaucheur JP, Drouot X, Ménard-Lefaucheur I, Keravel Y, Nguyen JP (2006). Motor cortex rTMS restores defective intra-cortical inhibition in chronic neuropathic pain. Neurology.

[R119] Lefaucheur JP, Hatem S, Nineb A, Ménard-Lefaucheur I, Wendling S, Keravel Y, Nguyen JP (2006). Somatotopic organization of the analgesic effects of motor cortex rTMS in neuropathic pain. Neurology.

[R120] Lefaucheur JP, Ménard-Lefaucheur I, Goujon C, Keravel Y, Nguyen JP (2011). Predictive value of rTMS in the identification of responders to epidural motor cortex stimulation therapy for pain. Journal of Pain.

[R121] Lenoir C, Algoet M, Mouraux A (2018). Deep continuous theta burst stimulation of the operculo-insular cortex selectively affects A*δ*-fibre heat pain. The Journal of Physiology.

[R122] Leung A, Shirvalkar P, Chen R, Kuluva J, Vaninetti M, Bermudes R, Poree L, Wassermann EM, Kopell B, Levy R (2020). Transcranial magnetic stimulation for pain, headache, and comorbid depression: INS-NANS expert consensus panel review and recommendation. Neuromodulation: Journal of the International Neuromodulation Society.

[R123] Liberati G, Algoet M, Santos SF, Ribeiro-Vaz JG, Raftopoulos C, Mouraux A (2019). Tonic thermonociceptive stimulation selectively modulates ongoing neural oscillations in the human posterior insula: Evidence from intracerebral EEG. NeuroImage.

[R124] Lindholm P, Lamusuo S, Taiminen T, Pesonen U, Lahti A, Virtanen A, Forssell H, Hietala J, Hagelberg N, Pertovaara A, Parkkola R (2015). Right secondary somatosensory cortex-a promising novel target for the treatment of drug-resistant neuropathic orofacial pain with repetitive transcranial magnetic stimulation. Pain.

[R125] Lisman J, Buzsáki G (2008). A neural coding scheme formed by the combined function of gamma and theta oscillations. Schizophrenia Bulletin.

[R126] Llinás RR (1988). The intrinsic electrophysiological properties of mammalian neurons: Insights into central nervous system function. Science.

[R127] Ma SM, Ni JX, Li XY, Yang LQ, Guo YN, Tang YZ (2015). High-frequency repetitive transcranial magnetic stimulation reduces pain in Postherpetic neuralgia. Pain Medicine (United States).

[R128] Marzouk T, Winkelbeiner S, Azizi H, Malhotra AK, Homan P (2020). Transcranial magnetic stimulation for positive symptoms in schizophrenia: A systematic review. Neuropsychobiology.

[R129] Mertens M, King OC, Van Putten MJAM, Boenink M (2022). Can we learn from hidden mistakes? Self-fulfilling prophecy and responsible neuroprognostic innovation extended essay. Journal of Medical Ethics.

[R130] Mhalla A, Baudic S, de Andrade DC, Gautron M, Perrot S, Teixeira MJ, Attal N, Bouhassira D (2011). Long-term maintenance of the analgesic effects of transcranial magnetic stimulation in fibromyalgia. Pain.

[R131] Moisset X, Bouhassira D, Attal N (2021). French guidelines for neuropathic pain: An update and commentary. Revue Neurologique.

[R132] Moisset X, de Andrade DC, Bouhassira D (2016). From pulses to pain relief: An update on the mechanisms of rTMS-induced analgesic effects. In European Journal of Pain.

[R133] Moisset X, Goudeau S, Poindessous-Jazat F, Baudic S, Clavelou P, Bouhassira D (2015). Prolonged continuous theta-burst stimulation is more analgesic than “classical” high frequency repetitive transcranial magnetic stimulation. Brain Stimulation.

[R134] Moisset X, Pereira B, Ciampi de Andrade D, Fontaine D, Lantéri-Minet M, Mawet J (2020). Neuromodulation techniques for acute and preventive migraine treatment: A systematic review and meta-analysis of randomized controlled trials. Journal of Headache and Pain.

[R135] Mori N, Hosomi K, Nishi A, Oshino S, Kishima H, Saitoh Y (2022). Analgesic effects of repetitive transcranial magnetic stimulation at different stimulus parameters for neuropathic pain: A randomized study. Neuromodulation.

[R136] Mueller K, Jech R, Růžička F, Holiga Š, Ballarini T, Bezdicek O, Möller HE, Vymazal J, Růžička E, Schroeter ML, Urgošík D (2018). Brain connectivity changes when comparing effects of subthalamic deep brain stimulation with levodopa treatment in Parkinson’s disease. NeuroImage: Clinical.

[R137] Mussigmann T, Bardel B, Lefaucheur JP (2022). Resting-state electroencephalography (EEG) biomarkers of chronic neuropathic pain. A Systematic Review NeuroImage.

[R138] Mylius V, Ayache SS, Ahdab R, Farhat WH, Zouari HG, Belke M, Brugières P, Wehrmann E, Krakow K, Timmesfeld N, Schmidt S (2013). Definition of DLPFC and M1 according to anatomical landmarks for navigated brain stimulation: Inter-rater reliability, accuracy, and influence of gender and age. NeuroImage.

[R139] Nahmias F, Debes C, de Andrade DC, Mhalla A, Bouhassira D (2009). Diffuse analgesic effects of unilateral repetitive transcranial magnetic stimulation (rTMS) in healthy volunteers. Pain.

[R140] Nardone R, Höller Y, Langthaler PB, Lochner P, Golaszewski S, Schwenker K, Brigo F, Trinka E (2017). RTMS of the prefrontal cortex has analgesic effects on neuropathic pain in subjects with spinal cord injury. Spinal Cord.

[R141] Neumann FE (1846). Allgemeine Gesetze der inducirten elektrischen Ströme. Annalen der Physik.

[R142] Niedermeyer E, Niedermeyer E, Lopes da Silva F (1999). Electroencephalography: Basic principles, clinical applications and related fields.

[R143] Nurmikko T, MacIver K, Bresnahan R, Hird E, Nelson A, Sacco P (2016). Motor cortex reorganization and repetitive transcranial magnetic stimulation for pain-a methodological study. Neuromodulation: Journal of the International Neuromodulation Society.

[R144] O’Connell NE, Marston L, Spencer S, Desouza LH, Wand BM (2018). Non-invasive brain stimulation techniques for chronic pain. Cochrane Database of Systematic Reviews.

[R145] Ojala J, Vanhanen J, Harno H, Lioumis P, Vaalto S, Kaunisto MA, Putaala J, Kangasniemi M, Kirveskari E, Mäkelä JP, Kalso E (2022). A randomized, sham-controlled trial of repetitive transcranial magnetic stimulation targeting M1 and S2 in central Poststroke pain: A pilot trial. Neuromodulation.

[R146] Okazaki YO, Nakagawa Y, Mizuno Y, Hanakawa T, Kitajo K (2021). Frequency- and area-specific phase entrainment of intrinsic cortical oscillations by repetitive transcranial magnetic stimulation. Frontiers in Human Neuroscience.

[R147] O’Reardon JP, Solvason HB, Janicak PG, Sampson S, Isenberg KE, Nahas Z, Mcdonald WM, Avery D, Fitzgerald PB, Loo C, Demitrack MA (2007). Efficacy and safety of transcranial magnetic stimulation in the acute treatment of major depression: A multisite randomized controlled trial.

[R148] Owens AP, Allen M, Ondobaka S, Friston KJ (2018). Interoceptive inference: From computational neuroscience to clinic. Neuroscience and Biobehavioral Reviews.

[R149] Pascual-leone A, Valls-solé J, Wassermann EM, Hallett M (1994). Responses to rapid-rate transcranial magnetic stimulation of the human motor cortex. Brain: A Journal of Neurology.

[R150] Passard A, Attal N, Benadhira R, Brasseur L, Saba G, Sichere P, Perrot S, Januel D, Bouhassira D (2007). Effects of unilateral repetitive transcranial magnetic stimulation of the motor cortex on chronic widespread pain in fibromyalgia. Brain: A Journal of Neurology.

[R151] Pei Q, Zhuo Z, Jing B, Meng Q, Ma X, Mo X, Liu H, Liang W, Ni J, Li H (2019). The effects of repetitive transcranial magnetic stimulation on the whole-brain functional network of postherpetic neuralgia patients. Medicine (Baltimore).

[R152] Peyron R, Quesada C, Fauchon C (2019). Handbook of clinical neurology.

[R153] Pfurtscheller G, Lopes Da Silva FH (1999). Event-related EEG/MEG synchronization and desynchronization: Basic principles. Clinical Neurophysiology: Official Journal of the International Federation of Clinical Neurophysiology.

[R154] Pommier B, Quesada C, Fauchon C, Nuti C, Vassal F, Peyron R (2018). Added value of multiple versus single sessions of repetitive transcranial magnetic stimulation in predicting motor cortex stimulation efficacy for refractory neuropathic pain. Journal of Neurosurgery.

[R155] Pretalli JB, Nicolier M, Chopard G, Vandel P, Tio G, Monnin J, Pazart L, Sechter D, Haffen E (2012). Resting motor threshold changes and clinical response to prefrontal repetitive transcranial magnetic stimulation in depressed patients. Psychiatry and Clinical Neurosciences.

[R156] Quesada C, Pommier B, Fauchon C, Bradley C, Créac’H C, Murat M, Vassal F, Peyron R (2020). New procedure of high-frequency repetitive transcranial magnetic stimulation for central neuropathic pain: A placebo-controlled randomized crossover study. Pain.

[R157] Rădulescu I, Drăgoi A, Trifu S, Cristea M (2021). Neuroplasticity and depression: Rewiring the brain’s networks through pharmacological therapy (review). Experimental and Therapeutic Medicine.

[R158] Rantamäki T, Yalcin I (2016). Antidepressant drug action—From rapid changes on network function to network rewiring. Progress in Neuro-Psychopharmacology and Biological Psychiatry.

[R159] Rosanova M, Casali A, Bellina V, Resta F, Mariotti M, Massimini M (2009). Natural frequencies of human corticothalamic circuits. The Journal of Neuroscience: The Official Journal of the Society for Neuroscience.

[R160] Rossini PM, Burke D, Chen R, Cohen LG, Daskalakis Z, di Iorio R, di Lazzaro V, Ferreri F, Fitzgerald PB, George MS, Hallett M (2015). Non-invasive electrical and magnetic stimulation of the brain, spinal cord, roots and peripheral nerves: Basic principles and procedures for routine clinical and research application: An updated report from an I. F.C.N. Committee. Clinical Neurophysiology.

[R161] Sacco P, Prior M, Poole H, Nurmikko T (2014). Repetitive transcranial magnetic stimulation over primary motor vs non-motor cortical targets; effects on experimental hyperalgesia in healthy subjects.

[R162] Saghazadeh A, Esfahani SA, Rezaei N (2016). Genetic polymorphisms and the adequacy of brain stimulation: State of the art. Expert Review of Neurotherapeutics.

[R163] Saitoh Y, Hirayama A, Kishima H, Oshino S, Hirata M, Kato A, Yoshimine T (2006). Stimulation of primary motor cortex for intractable deafferentation pain. Acta Neurochirurgica.

[R164] Salmelin R, Hämäläinen M, Kajola M, Hari R (1995). Functional segregation of movement-related rhythmic activity in the human brain. NeuroImage.

[R165] Sampson SM, Rome JD, Rummans TA (2006). Slow-frequency rTMS reduces fibromyalgia pain. Pain Medicine (Malden Mass).

[R166] Sampathkumar V, Miller-Hansen A, Sherman SM, Kasthuri N (2021). Integration of signals from different cortical areas in higher order thalamic neurons. Proceedings of the National Academy of Sciences of the United States of America.

[R167] Sarnthein J, Jeanmonod D (2008). High thalamocortical theta coherence in patients with neurogenic pain. NeuroImage.

[R168] Scarpetta S, Zhaoping L, Hertz J (2002). Hebbian imprinting and retrieval in oscillatory neural networks. Neural Computation.

[R169] Schaworonkow N, Triesch J, Ziemann U, Zrenner C (2019). EEG-triggered TMS reveals stronger brain state-dependent modulation of motor evoked potentials at weaker stimulation intensities. Brain Stimulation.

[R170] Seth AK, Friston KJ (2016). Active interoceptive inference and the emotional brain. Philosophical Transactions of the Royal Society B: Biological Sciences.

[R171] Short EB, Borckardt JJ, Anderson BS, Frohman H, Beam W, Reeves ST, George MS (2011). Ten sessions of adjunctive left prefrontal rTMS significantly reduces fibromyalgia pain: A randomized, controlled pilot study. Pain.

[R172] Siebner HR, Peller M, Willoch F, Minoshima S, Boecker H, Auer C, Drzezga A, Conrad B, Bartenstein P (2000). Lasting cortical activation after repetitive TMS of the motor cortex: A glucose metabolic study. Neurology.

[R173] Speer AM, Willis MW, Herscovitch P, Daube-Witherspoon M, Shelton JR, Benson BE, Post RM, Wassermann EM (2003). Intensity-dependent regional cerebral blood flow during 1-Hz repetitive transcranial magnetic stimulation (rTMS) in healthy volunteers studied with H215O positron emission tomography: I. effects of primary motor cortex rTMS. Biological Psychiatry.

[R174] Sporns O (2013). Structure and function of complex brain networks. Dialogues in Clinical Neuroscience.

[R175] OSPORNSter Braack EM, de Goede AA, van Putten MJAM (2019). Resting motor threshold, MEP and TEP variability during day-time. Brain Topography.

[R176] Tremblay S, Rogasch NC, Premoli I, Blumberger DM, Casarotto S, Chen R, Di Lazzaro V, Farzan F, Ferrarelli F, Fitzgerald PB, Hui J (2019). Clinical utility and prospective of TMS-EEG. Clinical neurophysiology.

[R177] Tsubokawa T, Katayama Y, Yamamoto T, Hirayama T, Koyama S (1991). Chronic motor cortex stimulation for the treatment of central pain. Acta Neurochirurgica Supplementum.

[R178] Tsubokawa T, Katayama Y, Yamamoto T, Hirayama T, Koyama S (1993). Chronic motor cortex stimulation in patients with thalamic pain. Journal of Neurosurgery.

[R179] Tzabazis A, Aparici CM, Rowbotham MC, Schneider MB, Etkin A, Yeomans DC (2013). Shaped magnetic field pulses by multi-coil repetitive transcranial magnetic stimulation (rTMS) differentially modulate anterior cingulate cortex responses and pain in volunteers and fibromyalgia patients. Molecular Pain.

[R180] Uhm KE, Kim YH, Yoon KJ, Hwang JM, Chang WH (2015). BDNF genotype influence the efficacy of rTMS in stroke patients. Neuroscience Letters.

[R181] Vetterlein A, Monzel M, Reuter M (2023). Are catechol-O-methyltransferase gene polymorphisms genetic markers for pain sensitivity after all?—A review and meta-analysis. Neuroscience and Biobehavioral Reviews.

[R182] Zarkowski P, Navarro R, Pavlicova M, George MS, Avery D (2009). The effect of daily prefrontal repetitive transcranial magnetic stimulation over several weeks on resting motor threshold. Brain Stimulation.

[R183] Zhang X, Hu Y, Tao W, Zhu H, Xiao D, Li Y (2017). The effect of motor cortex stimulation on central Poststroke pain in a series of 16 patients with a mean follow-up of 28 months. Neuromodulation: Journal of the International Neuromodulation Society.

[R184] Zhao CG, Sun W, Ju F, Wang H, Sun XL, Mou X, Yuan H (2020). Analgesic effects of directed repetitive transcranial magnetic stimulation in acute neuropathic pain after spinal cord injury. Pain medicine (Malden Mass).

[R185] Zhao C-G, Wei S, Fen J, Shan J, Hong W, Xiang X-LS, Yuan MH (2021). analgesic effects of navigated repetitive transcranial magnetic stimulation in patients with acute central Poststroke pain. Pain and Therapy.

[R186] Zrenner C, Desideri D, Belardinelli P, Ziemann U (2018). Real-time EEG-defined excitability states determine efficacy of TMS-induced plasticity in human motor cortex. Brain Stimulation.

